# T-cell activation by treatment of cancer patients with EMD 521873 (Selectikine), an IL-2/anti-DNA fusion protein

**DOI:** 10.1186/1479-5876-11-5

**Published:** 2013-01-07

**Authors:** Julien Laurent, Cedric Touvrey, Silke Gillessen, Magali Joffraud, Manuela Vicari, Caroline Bertrand, Stefano Ongarello, Bernd Liedert, Elisa Gallerani, Joachim Beck, Aurelius Omlin, Cristiana Sessa, Sonia Quaratino, Roger Stupp, Ulrike S Gnad-Vogt, Daniel E Speiser

**Affiliations:** 1 Division of Experimental Oncology, Multidisciplinary Oncology Center, Centre Hospitalier Universitaire Vaudois, Lausanne, Switzerland; 2 Kantonsspital, St. Gallen, Switzerland; 3 Global Clinical Development Unit-Oncology, Merck KGaA, Frankfurter Str. 250, D-64293, Darmstadt, Germany; 4 Oncology Institute of Southern Switzerland, Bellinzona, Switzerland; 5 University of Mainz, Mainz, Germany; 6 Multidisciplinary Oncology Center, Centre Hospitalier Universitaire Vaudois and University of Lausanne, Lausanne, Switzerland; 7 Clinical Tumor Biology & Immunotherapy Unit, Ludwig Center of the University of Lausanne, Lausanne, Switzerland; 8 IRBA antenne CRSSA, La Tonche, France; 9 CureVac GmbH, Frankfurt, Germany

**Keywords:** Interleukin-2, Fusion protein, Tumor, Lymphocytes, Cancer-testis antigen

## Abstract

**Background:**

EMD 521873 (Selectikine or NHS-IL2LT) is a fusion protein consisting of modified human IL-2 which binds specifically to the high-affinity IL-2 receptor, and an antibody specific for both single- and double-stranded DNA, designed to facilitate the enrichment of IL-2 in tumor tissue.

**Methods:**

An extensive analysis of pharmacodynamic (PD) markers associated with target modulation was assessed during a first-in-human phase I dose-escalation trial of Selectikine.

**Results:**

Thirty-nine patients with metastatic or locally advanced tumors refractory to standard treatments were treated with increasing doses of Selectikine, and nine further patients received additional cyclophosphamide. PD analysis, assessed during the first two treatment cycles, revealed strong activation of both CD4^+^ and CD8^+^ T-cells and only weak NK cell activation. No dose response was observed. As expected, Treg cells responded actively to Selectikine but remained at lower frequency than effector CD4^+^ T-cells. Interestingly, patient survival correlated positively with both high lymphocyte counts and low levels of activated CD8^+^ T-cells at baseline, the latter of which was associated with enhanced T-cell responses to the treatment.

**Conclusions:**

The results confirm the selectivity of Selectikine with predominant T-cell and low NK cell activation, supporting follow-up studies assessing the clinical efficacy of Selectikine for cancer patients.

## Background

T-lymphocytes have the potential to limit tumor progression but may be impaired in their function due to lack of growth factors and/or the presence of immunosuppressive mechanisms, particularly within metastatic lesions [1-3]. Immunotherapy with recombinant human interleukin-2 (IL-2) is an attractive treatment option for certain metastatic cancers, as it exerts both stimulatory and regulatory functions on the immune system and is, along with other members of the common γ chain (γc) cytokine family, central to immune homeostasis [4,5].

IL-2 acts via IL-2 receptors (IL-2R), consisting of either the trimeric αβγ receptor, or the dimeric βγ receptor [6]. Both IL-2R variants transmit signaling upon IL-2 binding. However, the trimeric αβγ receptor has 10–100 times higher affinity for IL-2 than the dimeric form [7], because IL-2Rα (CD25) confers high-affinity binding to IL-2. The trimeric IL-2R is mainly expressed on activated T-cells and CD4^+^ Treg cells (forkhead box P3 [Foxp3] positive T-cells) [7]. High-dose IL-2 is used for the treatment of patients with metastatic melanoma and metastatic renal cell carcinoma, and has a long-term impact on overall survival [8,9]. However, high-dose IL-2 treatment is associated with considerable toxicity, in particular vascular leak syndrome (VLS) with accumulation of extravascular fluid in organs such as the lung and liver. [10-12]. There is no treatment for VLS other than withdrawal of IL-2. Low-dose IL-2 regimens have been tested in patients to reduce side effects, at the price of reduced therapeutic results [13,14]. Many mechanisms have been proposed to explain VLS. Toxicity has been attributed to direct binding of IL-2 to endothelial cells via a motif resembling a component of bacterial toxins [15] and centered around aspartic acid residue 20 (D20); others reported a vasopermeability enhancing fragment of IL-2 extending from residues 22 to 58 that increases vascular permeability independent of IL-2 bioactivity [10], or proposed that activation of cells bearing the intermediate-affinity IL-2 receptor in the vascular compartment leads to inflammatory cytokine release by natural killer (NK) and other cells [16]. In order to target IL-2 to tumor in a way of reducing its potential toxicity, Merck KGaA (Darmstadt, Germany) developed a novel fully humanized IL-2 fusion protein, EMD521873 or NHS-IL2LT (Selectikine), for the treatment of solid tumors and B-cell non-Hodgkin lymphoma [17]. Selectikine comprises the monoclonal antibody (mAb) NHS76, which recognizes single-or double-stranded DNA often released from dying tumor cells either spontaneously or following treatment with radiation or chemotherapy [18,19], and two genetically modified IL-2 molecules, with a D20T mutation aimed at eliminating the toxin motif responsible for endothelial cell binding [15]. Pre-clinical data revealed that Selectikine retained low toxicity and induced anti-tumor responses. Multiple cycles of treatment can be administered safely and with the potential for improved efficacy. Furthermore, it has been shown that the D20T mutation, in the context of a whole antibody immunocytokine, was highly selective for the high-affinity IL-2R [17]. A caveat of targeting the high-affinity receptor is the possibility of activating Treg cells. Selectikine was tested in a phase I dose-escalation first-in-human study in patients with advanced solid tumors, as a 1-h intravenous (iv) infusion on 3 consecutive days every 3 weeks (group 1). Low-dose administration of cyclophosphamide at 300 mg/m^2^ one day prior to the first Selectikine infusion in each cycle was also assessed (group 2) as cyclophosphamide has been reported to suppress Treg cells and to enhance the antitumor activity of immunotherapy [20-23]. At all dose levels tested, no severe cardiovascular side-effects including severe hypotension or vascular leak syndrome, usually associated with native IL-2, were observed. Also, no objective tumor responses, but prolonged periods of disease stabilization in some patients, were observed [24]. We took the opportunity of this first-in-human phase I clinical trial to investigate immune modulatory effects induced by Selectikine. To this aim, an extensive analysis of pharmacodynamic markers was conducted during the first two treatment cycles. Here we report the results of this immune-monitoring, mainly from patients treated in group I.

## Materials and methods

Study design, patient eligibility criteria and clinical data are detailed in the Additional file 1 available online, and in the recent paper by Gillessen et al. [24]. The study was performed in accordance with the guidelines of the declaration of Helsinki, the International Conference on harmonization, and regulatory authorities and the protocol was approved by local ethics committees.

### Blood samples

For flow cytometry analysis, whole blood (10 mL) was collected in EDTA tubes (Vacutainer® Blood Collection Tubes, Becton-Dickinson [BD], Basel, Switzerland) on day 1, before Selectikine infusion (group 1) or before cyclophosphamide administration (group 2) and on day 8 during the first two cycles of treatment, and sent to a central laboratory for direct analysis within 24 h. During the second cycle, on days 1 and 8 for both patient groups, an additional 50 mL of blood was collected in Lithium-Heparin tubes (Vacutainer® Blood Collection Tubes, BD) and PBMC were isolated and frozen locally for further functional analysis. Briefly, blood was diluted 50% with PBS, overlayed onto Ficoll-Histopaque 1.077 (Sigma-Aldrich Chemie Gmbh, Munich, Germany) and centrifuged for 30 min at 400 × g and 20°C. The PBMC fraction was collected, washed in PBS, counted, aliquoted, and frozen at −80°C in 90% FCS/10% DMSO. In addition, 6 mL of whole blood was collected in Vacutainer SST tubes (BD) on days 1 and 3 during the first two cycles. Sera were prepared locally and frozen until analysis. Serum levels of IL-10, sIL-2R (R&D systems, Minneapolis, USA) and neopterin (IBL, Hamburg, Germany) were measured by ELISA according to the manufacturer’s instructions.

### Flow cytometry

Fluorescence-activated cell sorting (FACS) analysis on fresh blood was performed on total leucocytes after lysis of erythrocytes. Briefly, cells were stained with the following mAbs: FITC-conjugated anti-Bcl2, anti-Ki67, anti-perforin; and isotype control IgG1k or IgG2b; PE-conjugated anti-CD45RA, anti-CD127; PerCPconjugated anti-CD8, anti-CD3, anti-HLA-DR, anti-CD56 and anti-CD16; PE-Cy7-conjugated anti-CD25, anti-CCR7; APC-conjugated anti-CD38, anti-HLA-DR, anti-granzyme B, and isotype control IgG1k; APC-H7-conjugated anti-CD4 and anti-CD3. Antibodies were purchased from BD Pharmingen, except for FITC-conjugated anti-Foxp3 which was purchased from eBiosciences, San Diego, USA. Data were acquired using a FACS LSRII flow cytometry machine (BD), and analyzed with FCSExpress version 3 software (De Novo software, Ontario, Canada).

### Peptide stimulation assay

Antigen-specific CD8^+^ T-cell responses specific for cancer-testis tumor antigens were assessed *in vitro* by stimulating PBMCs collected on days 1 and 8 of the second cycle of Selectikine treatment with five different HLA-A2 restricted peptides (Melan-A/ELAGIGILTV; MAGE-A3/KVAELVHFL; NY-ESO-1/SLLMWITQA; MAGE-A10/GLYDGMEHL and SSX-2/KASEKIFYV). Briefly, CD8^+^ T-cells (1 × 10^5^) enriched by magnetic beads (Miltenyi Biotec, Bergisch Gladbach, Germany) were co-cultured with irradiated CD8^-^ cells (ratio of 1:1) in RPMI plus 8% AB human serum (Sigma-Aldrich Chemie GmbH, Buchs, Switzerland) in 96-well plates, and stimulated with the peptides (20 μM). After 48 h, medium was supplemented with IL-2 (150 U/mL) and IL-7 (20 ng/m). On day 7, cells were collected, stained with multimers and antibodies, and analyzed on an LSRII flow cytometer (BD). Acquired data were analyzed using FCSExpress version 3 software (De Novo software).

### Intracellular cytokine staining

Intracellular cytokine staining for IFNγ and TNFα was performed together with labeling with tetramers and CD8-specific antibodies. 1 × 10^6^ CD8^+^ enriched T-cells (Miltenyi Biotec) were incubated for 5 hours at 37°C with 1 × 10^6^ T2 cells pulsed with 10 μg/mL irrelevant HIV-1 Pol476–484 (ILKEPVHGV) peptide, or 10 μg/mL tumor antigenic peptides, or 1 μg/mL PMA/0.25 μg/mL ionomycin. After 1 h, 10 μg/mL brefeldin A (Sigma-Aldrich Chemie GmbH, Buchs, Switzerland) was added. 4 h later cells were stained with multimers and antibodies, fixed, permeabilized, and incubated with anti-IFNγ-FITC and anti-TNFα-APC mAbs in PBS/0.1% saponin for 30 min at 4°C. Cells were analyzed on a LSRII flow cytometer (BD). Acquired data were analyzed using FCSExpress version 3 software (De Novo software).

### Treg cell inhibition test

To test the suppressive activity of Treg cells, their ability to inhibit the proliferation of autologous CD4^+^ T-cells we measured in CFSE assays. Briefly, CD4^+^ T-cells were negatively selected using magnetic beads from PBMC collected on days 1 and 8 of the second cycle of treatment. The CD25-expressing CD4^+^ fraction was then positively selected using the CD4^+^CD25^+^ regulatory T-cell isolation kit (Miltenyi Biotec) according to the manufacturer’s instructions. CD4^+^CD25^-^ T-effector cells (6 × 10^5^) were labeled with CFSE (final concentration 2 μM) and cultured in 96 well-plates, stimulated with anti-CD3 and anti-CD28 beads (Dynabeads® Human T-Activator CD3/CD28, LuBioScience GmbH, Lucerne, Switzerland). CD4^+^CD25^+^ non-labeled Treg cells (3 × 10^5^) were added to the cultures (ratio of 1:2). After 4 days, cells were collected, and stained with the apoptotic marker VIVID, and anti-CD4 and anti-CD3 mAbs. CFSE intensity was measured on a FACS LSRII (BD).

### Immunohistochemistry

Tumor tissues from archival material (pretreatment) and a biopsy collected after two treatment cycles were analyzed by immunohistochemistry. Four-micrometer thick serial sections of formalin-fixed, paraffin-embedded tissue samples were prepared. Antigen retrieval carried out using microwave treatment in 0.1 M sodium citrate, pH 6.0. Staining was performed with anti-CD8, anti-CD4, anti-Foxp3 and anti-Ki67 mAbs. Detection was with the DAKO EnVision™ + system using diaminobenzidine (DAB) as the chromogen (DAKO, Trappes, France). Non-immune mouse IgG was used as a negative control. In parallel, tissue samples were stained with hematoxylin/eosin.

### Statistical analyses

Values are expressed as mean ± 95% confidence intervals. Statistical analysis was aimed at discovering differences due to the treatment, both in time and by dose level. Leukocyte subsets were compared between day 1 and 8 of the first two cycles using repeated measurements mixed model analysis of variance (ANOVA). The F-test, α = 0.05, in the ANOVA was used to test the fixed effects, and a post-hoc test (Tukey HSD) was applied for the pairwise comparisons. Differences were considered statistically significant at *P < 0.05 (**P ≤ 0.01, ***P ≤ 0.005). All analyses were performed using JMP v.8.0.2. software (SAS Institute Inc., Cary, US).

### Kaplan Meier survival analysis

Survival analyses were performed from the first day of study treatment to the date of progression, or date of death, or censored at the date of last observation. Kaplan Meier (KM) analyses were performed to estimate the relationships between survival time and biomarker baseline levels. For each biomarker individually, the baseline value for every patient was categorized into LOW or HIGH categories according to levels that were less or equal to the 33th percentile, or greater than the 66th percentile. The analysis was performed by the Unit for Bioinformatics (Merck Serono, Geneva), using the standard model: Overall Survival Time * Censoring. A log-rank test was performed to compare the two groups (LOW, HIGH). Kaplan Meier survival analysis was performed using JMP v.8.0.2. software (SAS Institute Inc., Cary, US).

## Results

### Clinical background

A total of 48 patients were enrolled, 39 were treated with Selectikine alone (group 1) in a 3 + 3 dose-escalation design starting from 0.075 mg/kg through 0.15, 0.225, 0.3, 0.45, 0.6 and finally 0.9 mg/kg; and nine patients with Selectikine plus cyclophosphamide (group 2, dose levels 0.45 mg/kg and 0.6 mg/kg). Skin rash at a dose of 0.9 mg/kg was the dose limiting toxicity, and the maximum tolerated dose was therefore determined as 0.6 mg/kg in group 1. The Selectikine treatment was associated with typical IL-2-like biological effects including lymphopenia followed by lymphocytosis and eosinophilia at all dose levels, while IL-2 related clinical AEs were mainly mild to moderate. The skin rash responded well to topical corticosteroids. Compared to previous studies, with intermediate to high IL-2 doses, Selectikine induced only mild hypotension and no vascular leak syndrome suggesting improved tolerability of this modified and more selective IL-2 moiety. Detailed clinical results have been reported in a recent publication [24].

In the following sections, we focus on the analysis of pharmacodynamic markers, mainly assessed during the first two treatment cycles; on day 1 before Selectikine infusion (day −1 for group 2), and on day 8.

### Leukocytes

**Figure 1:**
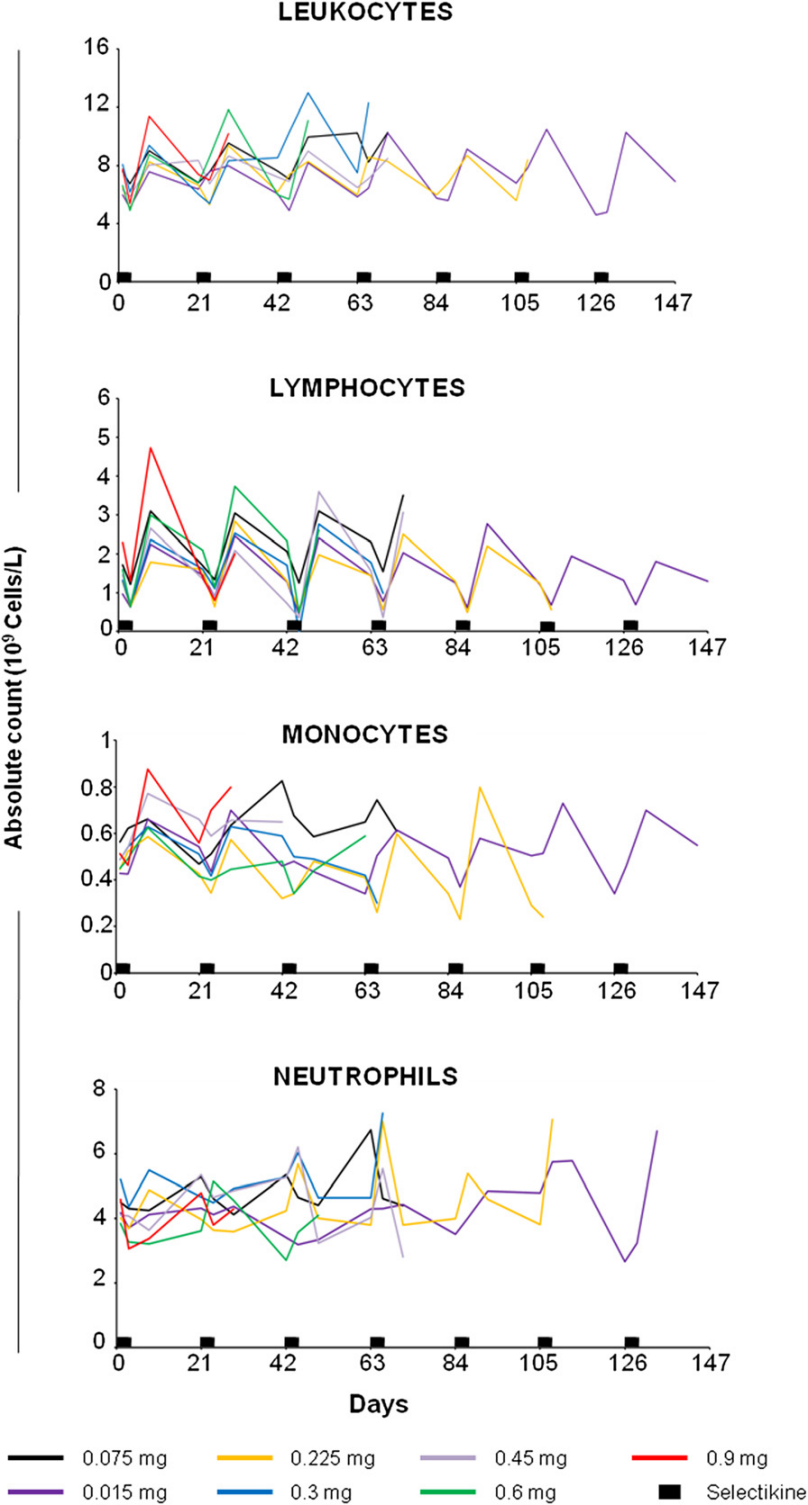
**Kinetics of subpopulations of leukocytes in patients treated with Selectikine.** Absolute counts (10^9 ^cells/L) of total leukocytes, lymphocytes, monocytes and neutrophils from patients treated at increasing doses of Selectikine (0.075, 0.15, 0.225, 0.3, 0.45, 0.6 and 0.9 mg/kg). Geometric mean values of absolute counts are shown for each dose-group, until discontinuation. Blood was drawn during each treatment cycle at the following time points: day 1 before start of Selectikine infusion and on day 3 and day 8 after start of Selectikine infusion. Black boxes on the x-axis represent the treatment, i.e. the 1-h iv infusions of Selectikine during three consecutive days per cycle.

Absolute counts of leukocyte populations in peripheral blood (clinical laboratory data), including lymphocytes, monocytes and neutrophils were measured for each cycle of treatment at the following time points: before the start of Selectikine infusion (day 1), on day 3 and day 8. Leukocytes were analyzed for the dose groups from 0.075 to 0.9 mg/kg Selectikine until treatment discontinuation. The data revealed lymphopenia at day 3 at all doses tested, followed by lymphocytosis (absolute leukocytes count) 8 days after Selectikine administration, with return to basal levels after each cycle (Figure 1, upper graph). Lymphocytes were most sensitive to Selectikine, with a systematic and steady increase between each cycle; whereas the responses of monocytes and neutrophils were less strong (Figure 1, lower graphs). Notably, no significant dose effects were observed between patients subgroups, therefore the data from the subgroups were pooled for the remaining analyses.

### CD4^+^ and CD8^+^ T-cells

**Figure 2:**
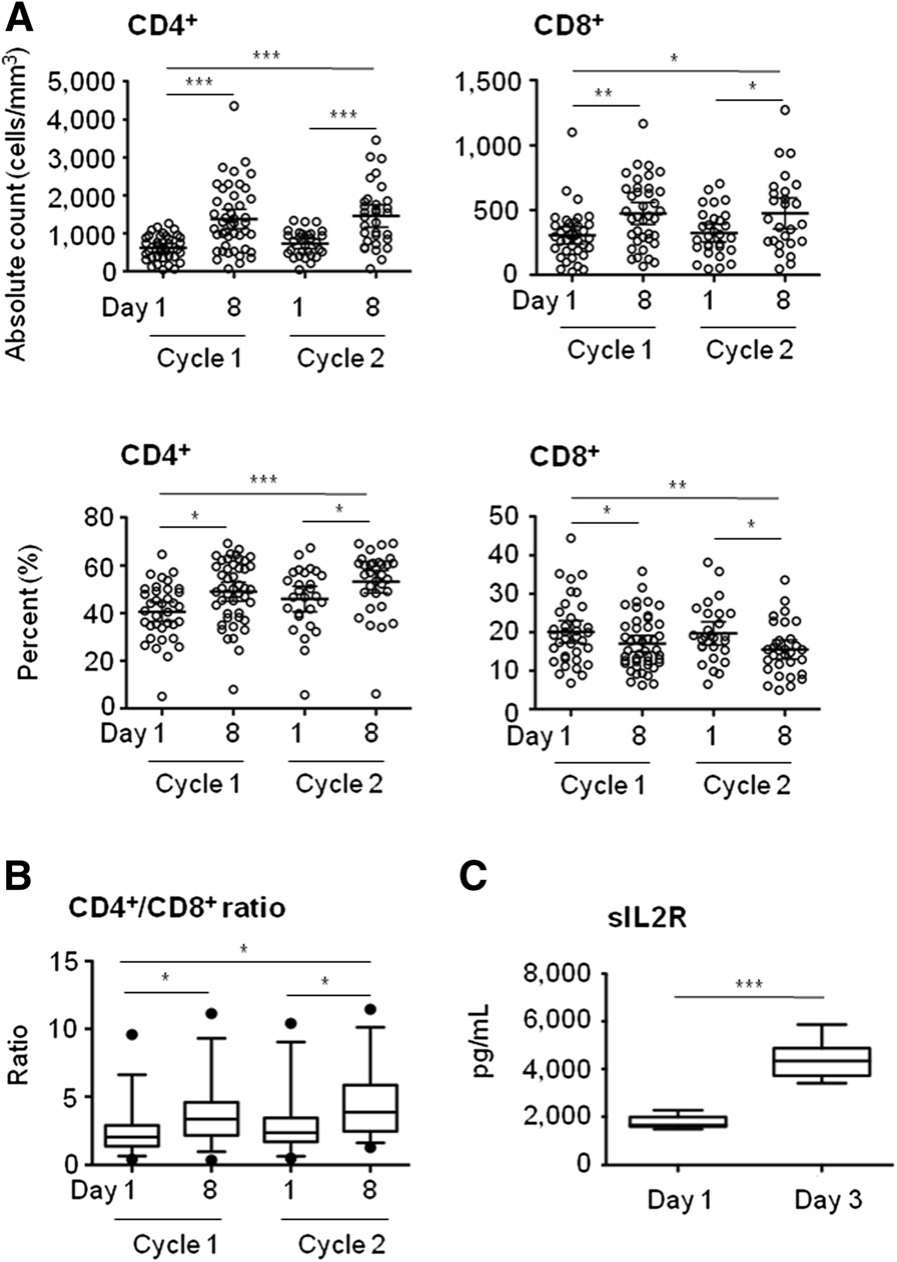
**Effects of Selectikine treatment on peripheral CD4**^**+ **^**and CD8**^**+ **^**T-cells. **(**A**) Absolute counts (cells/mm^3^) and frequencies (%) of CD3^+^CD4^+ ^and CD3^+^CD8^+ ^T-cells (gated on lymphocytes using FSC/SSC parameters) on days 1 and 8 during the first and second treatment cycles. Graphs show the individual values of 39 patients treated with 0.075 to 0.9 mg/kg Selectikine alone. Bars represent geometric mean values, with 95% CI. (**B**) CD4^+^/CD8^+ ^ratios on days 1 and 8 during the first and second treatment cycles. Box-and-whiskers graphs show geometric mean with 95% CI. (**C**) Serum concentrations (ng/mL) of soluble interleukin-2 receptor on days 1 and 3 during the first and second treatment cycle. The box and whisker graph shows geometric mean values per dose-group. *P < 0.05, **P ≤ 0.01, ***P ≤ 0.005.

As lymphocytes responded strongly to treatment, CD8^+^ and CD4^+^ T-cells were next analyzed during the first two treatment cycles. As expected, a statistically significant and transitory increase in absolute counts as well as in the frequency of both CD8^+^ and CD4^+^ T-cells was observed on day 8 after Selectikine infusion (Figure 2A). Notably, there was a greater increase in CD4^+^ T-cells on day 8 of both cycles (2.3-fold increase) compared with CD8^+^ T-cells (1.5-fold increase for cycles 1 and 2), leading to a relatively small but statistically significant increase of the CD4^+^/CD8^+^ ratio after both cycles of treatment (Figure 2B). As expected, serum concentrations of soluble IL-2R (sIL-2R) were also strongly increased shortly after Selectikine infusion (Figure 2C). The analysis of NK cells based on CD56 and CD16 surface markers showed no significant changes for both cycles monitored in terms of frequency or cytotoxicity (Additional file 1: Figure S1A and S1B), only a slight increase in absolute numbers was noted during the first cycle (Additional file 1: Figure S1A). No dose effects were observed in the analysis of NK cells.

### T-cell activation and proliferation

**Figure 3:**
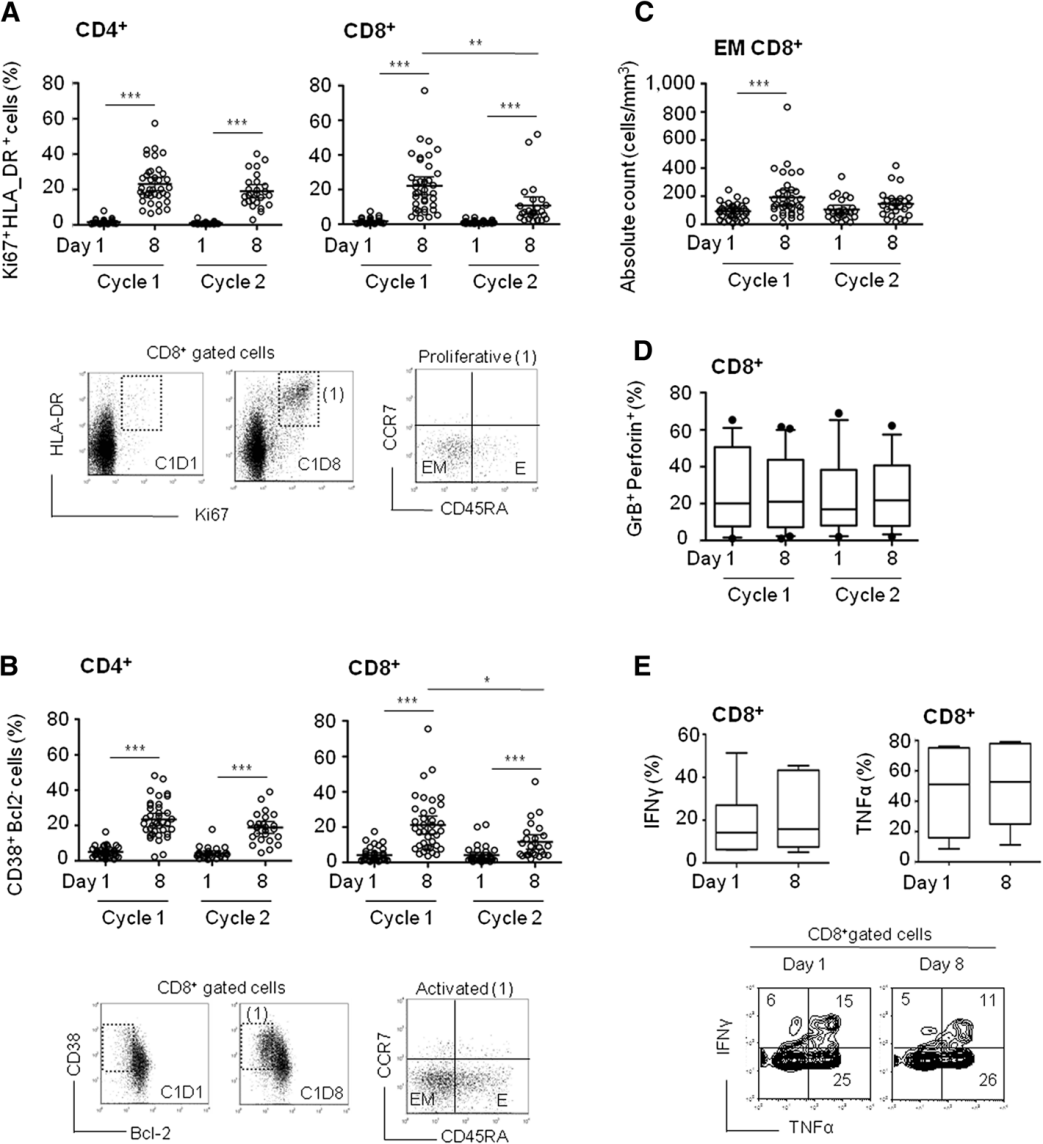
**Functional analysis of CD4**^**+ **^**and CD8**^**+ **^**T-cells before and after treatment with Selectikine. **(**A**) Frequencies (%) of proliferative CD4^+ ^and CD8^+ ^T-cells (Ki67^+ ^HLA-DR^+^) on days 1 and 8 during the first and second treatment cycles. Dot plots represent HLA-DR versus Ki67 staining of CD8^+ ^gated T-cells on days 1 and 8 during the first treatment cycle. The right dot plot shows expression of CCR7 and CD45RA of cells from gate (1), on day 8 of the first cycle. (**B**) Frequencies (%) of activated cells (CD38^+ ^Bcl2^-^) within CD4^+ ^and CD8^+^ T-cells on days 1 and 8 during the first and second treatment cycle. Dot plots represent CD38 versus Bcl2 staining in CD8^+ ^gated cells on days 1 and 8 during the first treatment cycle. The right dot plot represents CCR7 versus CD45RA repartition of activated CD8^+ ^T-cells (1) on day 8 of the first cycle. (**C**) Absolute counts (cells/mm^3^) of effector memory (EM) CD8^+^ T-cells (CCR7^- ^CD45RA^-^) on days 1 and 8 during the first and second treatment cycles. Graphs show individual values of 39 treated patients. Bars represent geometric mean values with 95% CI. (**D**) Frequency of GrB^+^ perforin^+ ^cells in CD8^+ ^gated cells on days 1 and 8 during the first and second treatment cycles. The box-and-whiskers graph shows geometric mean with 95% CI. (**E**) Expression of IFNγ and TNFα (%) in CD8^+ ^T-cells on days 1 and 8 of the second cycle, after a 4-hour *in vitro *stimulation with PMA/ionomycin. Dot plot representation of IFNγ versus TNFα staining in CD8^+ ^gated T-cells after in vitro stimulation. Box-and-whiskers graphs show geometric mean with 95% CI from 9 randomized patients treated with 0.075 to 0.9 mg/kg Selectikine alone. *P < 0.05, **P ≤ 0.01, ***P ≤ 0.005.

As CD8^+^ and CD4^+^ T-cells were both sensitive to Selectikine, the lymphocyte functions were next investigated. Combinations of Ki67- and HLA-DR-specific antibodies were used to analyze proliferation, and CD38- and Bcl2-specific antibodies for activation [25-27]. Strong proliferation was induced in both CD4^+^ and CD8^+^ T-cells after each treatment cycle, reaching a mean of approximately 20% of proliferative CD4^+^ T-cells in cycle 1 (23.1% ± 11.4) and 2 (19.0% ± 9.2). The proliferation of CD8^+^ T-cells was similar (22.2% ± 15.8) in the first cycle, but less in the second cycle (10.7% ± 12.4) (Figure 3A). Proliferating (HLA-DR^+^ Ki67^+^) cells were predominantly effector memory (EM) cells, as identified by the absence of CCR7 and CD45RA expression (Figure 3A dot plot, Additional file 1: Figure S2A). Similar to the proliferation state, activated cells (CD38^+^ Bcl-2^-^) were also increased after both cycles for CD4^+^ and CD8^+^ T-cells, with a decrease of the activation state of CD8^+^ T-cells during the second treatment cycle (Figure 3B). As expected, activated cells were primarily EM cells (Figure 3B dot plot, Additional file 1: Figure S2B). In accordance with these observations, the absolute count of EM CD4^+^ T-cells was increased during both cycles (Additional file 1: Figure S2C), whereas the absolute count of EM CD8^+^ T-cells only increased during the first cycle (Figure 3C). CD8^+^ effector cell properties were not found to be altered by Selectikine treatment, as indicated by *ex vivo* assessment of intracellular expression of granzyme B and perforin (Figure 3D), and the production of IFNγ and TNFα after a 4-h stimulation with PMA/ionomycin (Figure 3E). Finally, neopterin levels were increased, reflecting the overall activation of the cellular immune system (Additional file 1: Figure S2D).

### CD4^+^ Treg

**Figure 4:**
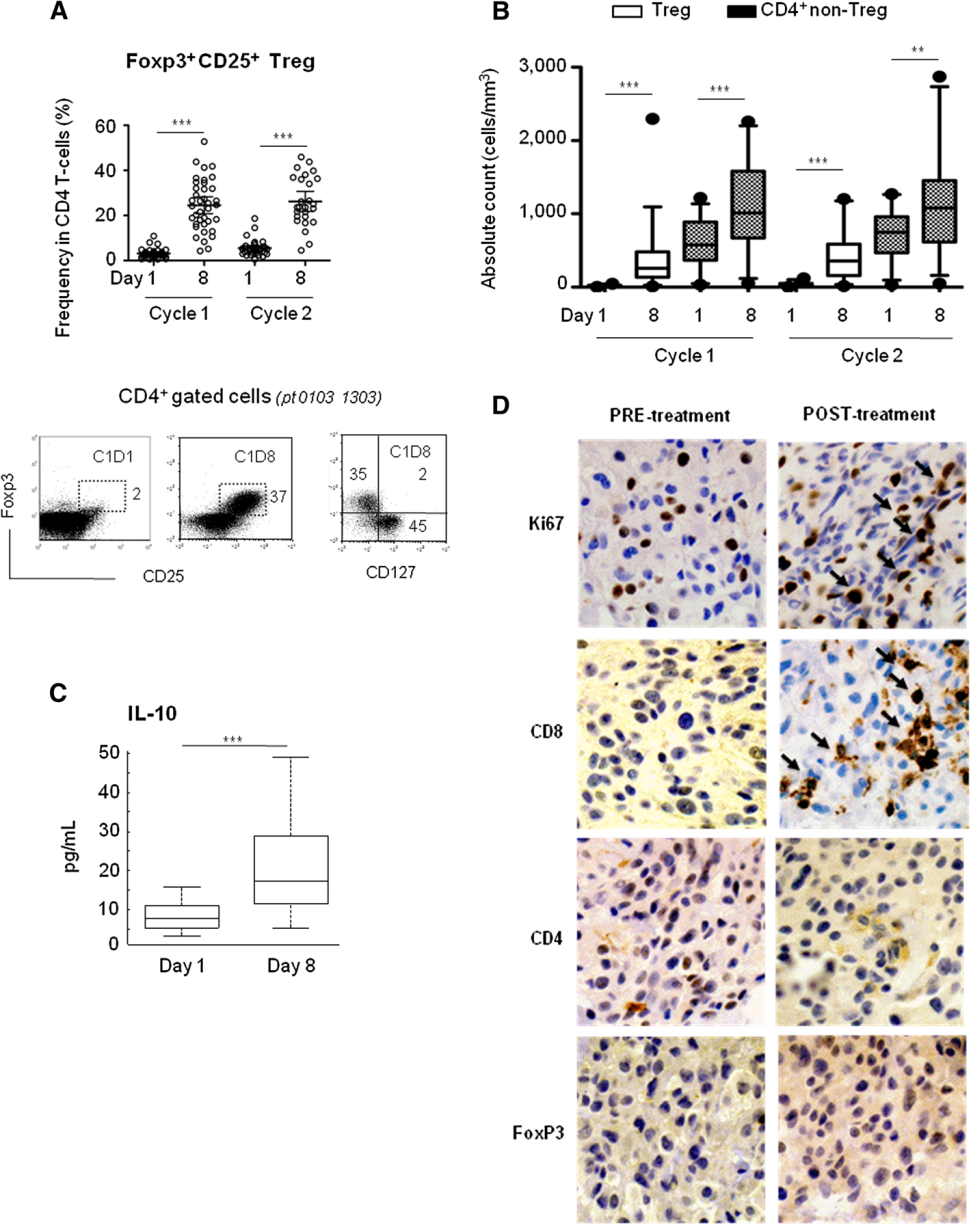
**Effects of Selectikine on regulatory CD4**^**+ **^**T-cells. **(**A**) Frequency (%) of CD4^+ ^Treg cells based on Foxp3 and CD25 expression. Dot plot representations of Foxp3 versus CD25 staining on days 1 and 8 during the first treatment cycle; and Foxp3 versus CD127 on day 8. Graphs represent individual values of 39 treated patients. Bars represent the geometric mean with 95% CI. (**B) **Absolute counts (Cells/mm3) of Treg cells (Foxp3^+^CD25^+^CD4^+^CD3^+^) and CD4^+ ^non-Treg cells (Foxp3^-^CD25^-^CD4^+^CD3^+^) on days 1 and 8 during the first and second treatment cycles. Box-and-whiskers graphs show geometric mean with 95% CI. (**C**) Serum concentrations (pg/mL) of IL-10 measured on days 1 and 3 after Selectikine infusion in 39 treated patients in patient group 1. (**D**) Ki67, CD8^+^, CD4^+ ^and Foxp3 immunoperoxydase staining in tumor tissue before the study (PRE-treatment), and after the second treatment cycle (POST-treatment) from melanoma patient N° 101 1102 treated with 0.225 mg/kg Selectikine. **P ≤ 0.01, ***P ≤ 0.005.

CD4^+^ Treg cells are inhibitory immune cells, characterized by high expression of Foxp3 and CD25. Besides CD25, Treg cells also express the remaining chains of the high-affinity IL-2 receptor, and are thus sensitive to IL-2 and Selectikine, similar to conventionally activated T-cells. As expected, the frequency of Treg cells was considerably increased after each treatment cycle, reaching a mean of approximately 25% of CD4^+^ T-cells (cycle 1, 24.5% ±11.3; cycle 2, 23.8% ±1.6) (Figure 4A). These Treg cells were CD3^+^ CD4^+^ CD25^+^ Foxp3^+^ and CD127^-^ (Figure 4A, dot plot); CD127 expression being mainly expressed on subpopulations of CD4^+^ non-Treg cells and CD8^+^ T-cells (Additional file 1: Figure S3A). Of note, a decrease in CD127 expression was observed on CD4^+^ T-cells but not on CD8^+^ T-cells. The strong sensitivity to Selectikine of Treg cells was confirmed by absolute counts, with 20 ± 60 and 11 ± 7-fold increase in cycles 1 and 2, respectively (day 8); while CD4^+^ non-Treg cells increased by 1.6 ± 5 and 1.5 ± 0.5-fold, respectively, similarly to CD8^+^ T-cells. This means that the CD4^+^/CD8^+^ ratio remained unchanged when excluding Treg cells (Figure 4B and Additional file 1: Figure S3B). Of note, a single injection of low-dose cyclophosphamide (group 2) did not affect their frequency (Additional file 1: Figure S3C). The inhibitory function of Treg cells at baseline (day 1) and on day 8 of the second treatment cycle was also measured in eight randomly selected patients from group 1 (Additional file 1: Figure S3D). In five of these patients, Treg cells inhibited CD4^+^ T-cell proliferation between 60 and 100% (ratio 1:2) to a similar extent on days 1 and 8. In the three remaining patients, Treg cells were not inhibitory on either day (Table 1). The increase of Treg cell frequency without changes of their inhibitory function may reflect early production of IL-10, as detected in sera on day 3 after the first dose of Selectikine (Figure 4C). Finally, analysis of Ki67 expression revealed that a mean of approximately 50% of Treg cells were proliferating cells on day 8 (cycle 1, 51.6% ±11.6; cycle 2, 46.7% ±15.8) (Additional file 1: Figure S3E).

**Table 1:** Treg inhibition capacity

**Inhibition of CD4**^**+ **^**proliferation by Tregs**			
**Dose**	**Patient**	**Inhibition (%)**	
		**Day 1**	**Day 8**
0.075	103 1301	99	88
0.075	103 1318	75	48
0.075	102 1213	68	49
0.3	103 1303	97	92
0.3	103 1309	0	0
0.6	103 1310	82	77
0.6	103 1314	0	2
0.6	101 1104	0	0

Capacity of inhibition (%) of CD4^+ ^T-cell proliferation induced by CD3^+^/CD28^+ ^stimulation by Treg cells, sorted on days 1 and 8 during the second treatment cycle. Samples from 8 randomized patients treated at 0.075, 0.3 and 0.6 mg/kg Selectikine alone were tested.

### In situ analysis

We further investigated whether lymphocytes accumulated *in situ*, by studying a post-treatment biopsy collected after the second cycle, from a patient with metastatic melanoma treated with 0.225 mg/kg of Selectikine and matched archival material collected shortly prior to start of Selectikine treatment. Interestingly immunohistochemistry staining revealed significantly increased expression of Ki67 by lymphocytes after Selectikine treatment compared with the pretreatment sample. Furthermore, there was an increased infiltration of CD8^+^ T-cells, while CD4^+^ and Foxp3^+^ Treg cells were unchanged (Figure 4D). Notably, post-therapy immune response in this patient was similar to other treated patients, with increased counts of CD8^+^, CD4^+^ and Treg cells, and strong activation and proliferation of T-cells (individual data not shown).

### Tumor-antigen specific CD8^+^ T-cells

We investigated whether Selectikine treatment promoted responses of tumor-antigen specific CD8^+^ T-cells. Of eight randomly selected HLA-A2 positive patients, CD8^+^ T-cells were isolated at baseline and on day 8 after Selectikine infusion during the second cycle, and stimulated with a panel of peptides representing cancer-testis antigenic epitopes from the tumor antigens Melan-A, MAGE-A3, MAGE-A10, NY-ESO-1 and SSX-2. After one week of peptide stimulation, cells were isolated and stained with tetramers combined with intracellular staining for IFNγ and TNFα. Lymphocytes obtained from day 8 (second treatment cycle) did not show significant enrichment of tumor-antigen specific T-cells compared with those from day 1 (Additional file 1: Figure S4A). One patient showed strong activation of Melan-A specific T-cells at the baseline of the second cycle, but without a further increase on day 8 (Additional file 1: Figure S4B). Measurements of IFNγ and TNFα production within tetramer positive CD8^+^ T-cells showed no functional enhancement after Selectikine treatment (Additional file 1: Figure S4C), which was similar to our findings for whole circulating CD8^+^ T-cells (Figure 3E).

### Correlation with patient survival

**Figure 5:**
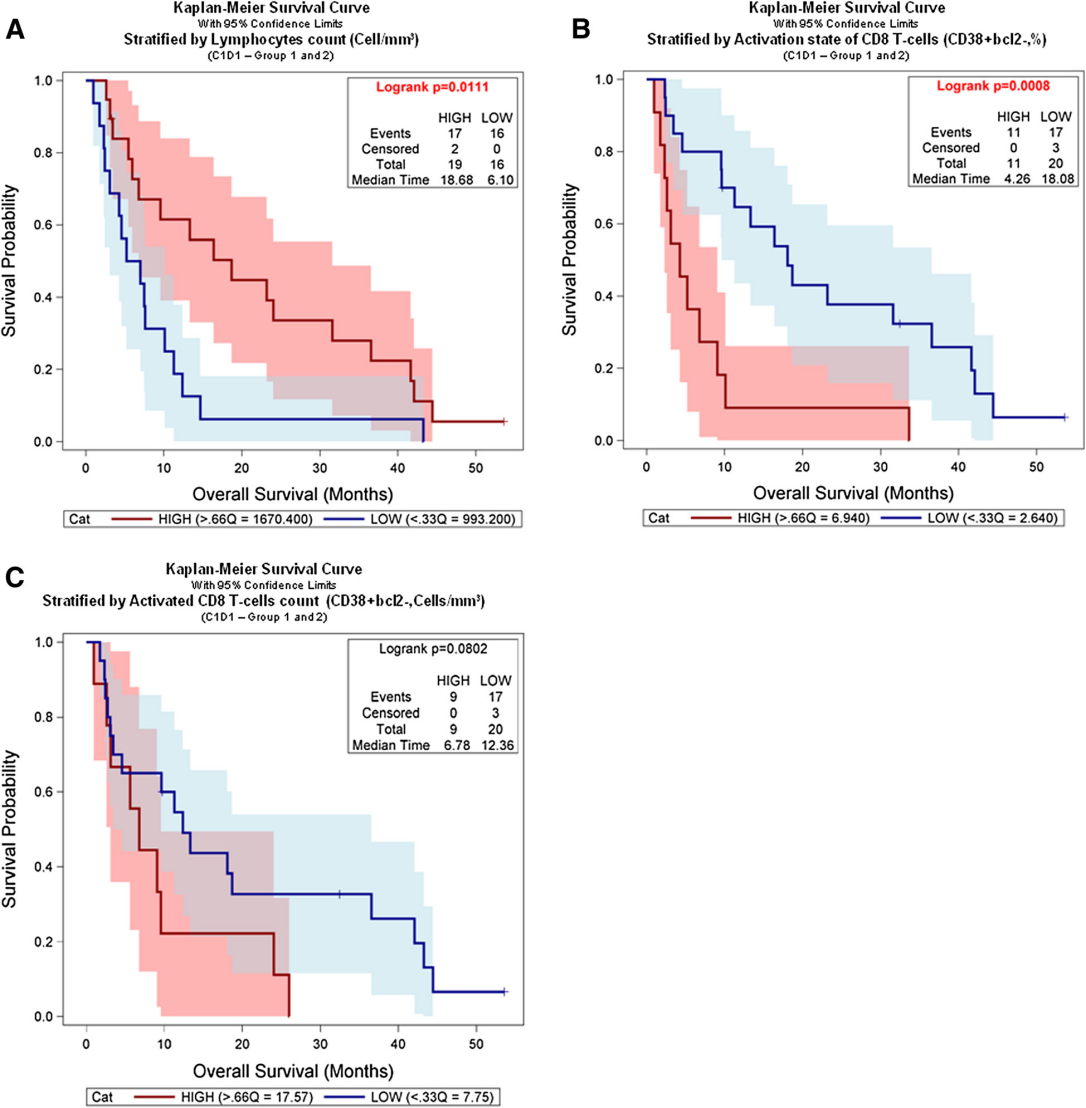
**Pharmacodynamic markers correlative evaluation with patient survival. **(**A**) Kaplan-Meier Survival Curve with 95% confidence limits stratified by lymphocyte counts (cell/mm^3^) at baseline in patient groups 1 and 2. (**B**) Kaplan-Meier Survival Curve with 95% confidence limits stratified by percent of activated CD8^+^ T-cells (CD38^+^bcl2^-^,%) at baseline in patient groups 1 and 2. (**C**) Kaplan-Meier Survival Curve with 95% confidence limits stratified by absolute count of activated CD8^+ ^T-cells (CD38^+^bcl2^-^, Cell/mm^3^) at baseline in patient groups 1 and 2.

Preliminary evidence of clinical efficacy was investigated. No tumor responses were recorded, but prolonged disease stabilization in some of these heavily pre-treated patients observed. Median overall survival was 9.6 months [95% CI 5.6–16.4] in group 1, and 7.0 months [95% CI 4.3–23.2] in group 2. Previously reported [28-30], associations between patient survival and lymphocyte properties (as described in Figures 1 and 2), were further examined. Interestingly, high lymphocyte counts at baseline were significantly associated with longer survival (Figure 5A). Furthermore low levels of activated CD8^+^ T-cells (CD38+Bcl2-%) at baseline were associated with longer patient survival (Figure 5B), while the absolute count was not (Figure 5C). Notably, the highest increase of CD8^+^ T-cell activation at day 8 was observed in patients who presented low activation levels before the start of treatment (Additional file 1: Figure S5A), and was thus independent of lymphocyte counts (Additional file 1: Figure S5B). Therefore, treatment induced T-cell activation appeared to occur preferentially in patients with low-level activation at baseline and seemed to be associated with improved clinical outcome in this small number of patients. Of note, Treg cells did not impact on overall survival (Additional file 1: Figure S5C).

## Discussion

IL-2 therapy can be effective in the treatment of patients with metastatic renal cell carcinoma and metastatic melanoma [31]. Clinical responses to high-dose IL-2 therapy occur in 15% to 20% of patients. However, side-effects are frequent and can be serious [32,33]. More targeted IL-2 therapies are necessary to improve safety and efficacy [34]. Several small phase II studies recorded some clinical responses with the administration of lower doses of IL-2 but no cases of long lasting responses were reported [35]. A randomized study comparing high- and low-dose IL-2 in 156 patients with metastatic renal cancer by Yang et al. [14], supported the finding that low-dose IL-2 regimens can cause the regression of advanced renal cell cancer. However, the higher dose of IL-2 appeared to produce greater biological activity, together with a higher clinical response rate.

The novel recombinant IL-2/anti-DNA fusion protein, Selectikine, was developed with the aim of maximizing immunomodulatory action and clinical efficacy at minimal vascular toxicity, despite treatment at optimal doses [36]. Our analysis of peripheral leukocytes showed a strong and transitory increase early after infusion of Selectikine. As expected, lymphocytes responded readily while the effects on monocytes were less pronounced, and no significant changes were observed to neutrophils (Figure 1). Strong increases in both percentages and absolute counts were observed for both CD4^+^ and CD8^+^ T-cells (Figure 2), while only weak effects were found for NK cells (Additional file 1: Figure S1), confirming the selectivity of Selectikine for T-cells compared with native IL-2 and an immunocytokine comprising wild-type IL-2 [37]. Furthermore, major biological effects included a significant increase in the soluble factors sIL-2R, neopterin and IL-10 [38]. Recently, treatment with EMD 273063, a humanized anti-GD2 mAb fused to native IL-2, showed no significant effects on peripheral CD4^+^ and CD8^+^ T-cells 10 days after infusion of the molecule [38]. Nevertheless, EMD 273063 administration resulted in increased levels of the soluble factors sIL-2R, neopterin and IL10, as reported in the present study.

Strong activation and proliferation of lymphocytes was observed, essentially of EM T-cells (Figure 3 and Additional file 1: Figure S2), even at the lowest dose (0.075 mg/kg) of Selectikine. In general, a dose effect on biological responses was not found. This was remarkable, since strong immune stimulation even at low doses of Selectikine, without major toxicity was demonstrated. In the field of IL-2 therapy, these biologic effects are considered as potentially beneficial. They are mostly observed at high doses with wild-type IL-2 and are thus associated with toxicity [39]. The absence of a dose effect in the present study should be interpreted with caution due to the heterogeneity of the patient population comprising different types of cancers with different pretreatment regimens. To reliably investigate a dose effect, a randomized study investigating different doses in a more homogeneous population would be necessary.

As expected, Treg cells responded actively, with increased frequencies and absolute counts after infusion of Selectikine (Figure 4A, B). However, their inhibitory capacities were unchanged (Table 1), based on the same cell ratio. The increase of Treg number is far more important than the increase of other T-cell populations, which could reflect a change in effector/regulatory T-cell ratio in vivo. Similarly to effector cells, Selectikine effects were transitory. Furthermore, increased Treg cell frequency appeared to be due to the proliferation of pre-existing Treg cells, as they expressed Ki67 (Additional file 1: Figure S3). The elevated levels of IL-10 may be a direct consequence of Treg cell sensitivity to the therapy (Figure 4C). Additional treatment with low dose cyclophosphamide prior to Selectikine infusion did not inhibit Treg cells (Additional file 1: Figure S3C), which is compatible with the majority of other studies [40]. The usefulness of cyclophosphamide for therapeutic inhibition of Treg cells remains questionable [41,42]. Importantly, in addition to Treg cells, the levels of CD4^+^ effector T-cells were also increased after the infusion of Selectikine (Figure 4B). Furthermore, CD8^+^ effector T-cells were also activated and increased in numbers and frequencies. Thus, even if Treg cells were highly sensitive to Selectikine, T-cell activation was broad, with increased activity of both effector and regulatory T-cells, in contrast to recent studies of low dose IL-2 treatment demonstrating preferential and sustained activation of Treg cells [43,44]. The fact that Selectikine targets the tumor tissue through the binding of free DNA may result in a different T-cell activation in the tissue compared to periphery, which could perhaps also be the case for Treg cells as suggested by the analysis of the single post-therapy biopsy (Figure 4D). For this patient, the in situ analysis revealed different effects in the tumor compared to the periphery. This aspect should be addressed in future clinical trials.

Recently, Levin et al. have published the first results evaluating a new class of IL-2 named a superkine, which has been engineered to eliminate the functional requirement of CD25 expression [45]. In vitro and in vivo experiments revealed that the new IL-2 superkine induced superior expansion of cytotoxic T-cells compared to Treg cells, leading to improved antitumor responses and reduced pulmonary edema. Compared with the observations made with Selectikine, it will be interesting to evaluate both molecules in terms of immunological responses, toxicity and VLS when the IL-2 superkine reaches the clinic.

PBMC available from before and after the second treatment cycle were studied to investigate whether Selectikine treatment had detectable effects on tumor antigen-specific CD8^+^ T-cells. Several patients presented detectable levels of tumor-specific T-cells, and one patient developed a strong response. However, in most patients there was no increase in the frequency or the functionality of tumor-specific T-cells (Additional file 1: Figure S4). Even though many of these T-cells may have been induced by the treatment, in the absence of the assessment of pretreatment samples, this could not be formally addressed in this study. Unfortunately, it was also not possible to determine patient tumor antigen expression, which made it impossible to exclude those T-cells with specificities for non-expressed antigens (i.e. the majority) from the analysis. As a first-in-human study of Selectikine, at the time of the study design, these factors were not of high priority.

In cancer patients, tumor-infiltrating lymphocyte counts and localization are often independent prognostic factors for survival [46-50]. However, several studies including our own (Figure 5A) showed also that the counts of circulating lymphocytes correlated positively with survival [28-30]. Notably, in our study enhanced pre-activation of CD8^+^ T-cells at baseline correlated with short survival (Figure 5B), while no correlation was observed when considering Treg cells (Additional file 1: Figure S5C). However, these are preliminary observations since the phase I study design and the low patient number do not allow for definitive conclusions on clinical outcome. Larger studies are required to address these questions, and to test whether low-level T-cell activation at baseline, and strong therapy induced T-cell activation are predictive for favourable clinical outcome.

## Conclusion

This first-in-human trial with Selectikine, a novel immunocytokine IL-2/anti-DNA fusion protein, confirms its selective biologic activity. Larger studies are now required to confirm our findings, and to determine potential clinical benefits. Finally, further analyses of post-treatment biopsies will provide more detailed insights into the biological effects of the dual targeting (IL-2R/DNA) by Selectikine.

## Abbreviations

DAB: Diaminobenzidine; EM: Effector memory; FACS: Fluorescence-activated cell sorting; IL-2: Interleukin-2; IL-2R: Interleukin-2 receptors; iv: Intravenous; mAb: Monoclonal antibody; PD: Pharmacodynamic.

## Competing interests

JL, SO, BL and SQ are currently employees of Merck KGaA and UG was an employee of Merck KGaA from 2005–2009, was a consultant for Merck KGaA from 2009–2011, and has received funds for travel from Merck KGaA. All other authors report no conflicts of interest.

## Authors’ contributions

JL participated in the design, analysis and data interpretation of immuno-monitoring and wrote the manuscript draft; CT, MV participated in FACs analysis; MJ performed in vitro functional tests and tetramer analysis; SO and BL performed analysis of soluble factors; SO participated in statistical analysis; SQ, RS were involved in study design; BL and UGV were medical leaders in the project; RS was involved in patient recruitment. All authors participated in critical review of the manuscript and final approval to submit.

## Supplementary Material

Additional file 1 Materials and methods. Click here for file
