# Supplementary material for: T-cell activation by treatment of cancer patients with EMD 521873 (Selectikine), an IL-2/anti-DNA fusion protein
Source: J Transl Med. 2013 Jan 7;11:5. doi: 10.1186/1479-5876-11-5 (PMC3551760; doi:10.1186/1479-5876-11-5)
Supplement: Additional file 1 — Materials and methods. [file 1479-5876-11-5-S1.pdf]

SUPPLEMENTARY INFORMATION

SUPPLEMENTARY MATERIALS AND METHODS

Study design

EMD 521873 (Selectikine), a newly developed IL-2/anti-DNA fusion protein was assessed in an open-label phase I study (EurdraCT number 2006-002083-26) in patients with metastatic or locally advanced tumors refractory to standard treatments. The study was performed in accordance with the guidelines of the declaration of Helsinki, the International Conference on harmonization, and regulatory authorities and the protocol was approved by local Ethics committees.

Main patient eligibility criteria

Eligible patients were 18 years or older, had an Eastern Cooperative Oncology Group (ECOG) performance status of 0 or 1 and histologically or cytologically proven metastatic or locally advanced solid tumors after failure of standard therapy. Exclusion criteria were IL-2 therapy within the last 6 months; radiotherapy, chemotherapy, major surgery or treatment with any investigational drug within the last 30 days. All patients gave written informed consent.

Treatment

Selectikine was administered as a 1-hour iv infusion on days 1-3 of a 21-day cycle (group 1). A second group of patients received 300 mg/m^2 CPA as a 1-hour iv infusion

one day before the first dose of Selectikine in each cycle (group 2). Selectikine was provided as a lyophilizate and reconstituted in sterile 0.9% sodium chloride for injection. In both groups, treatment was to be given until tumor progression or occurrence of intolerable side effects, or at the investigator's discretion as long as a clinical benefit was observed. Indomethacin or paracetamol was administered to all patients 2 hours before the first dose of Selectikine of each cycle, and then every 8 hours up to 3 days to alleviate flu-like symptoms.

A.

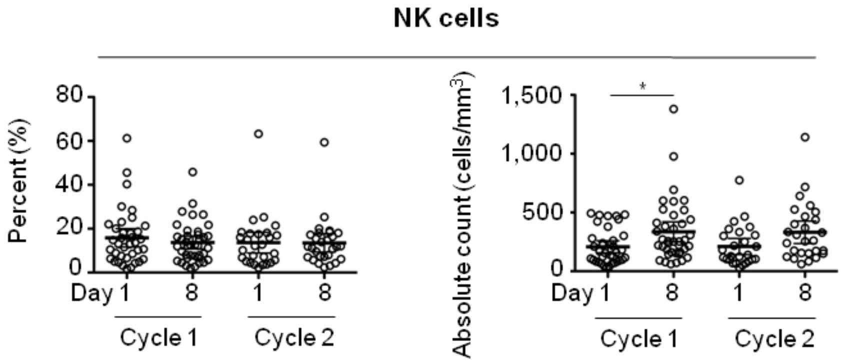

B.

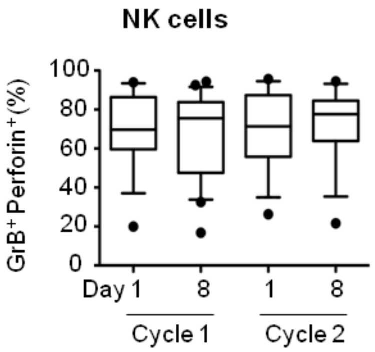

Supplementary Figure 1. Effect of Selectikine treatment on peripheral Natural killer (NK) cells. **A.** Frequencies (%) and absolute counts (cell/mm^3) of NK cells on days 1 and 8 during the first and second treatment cycles. Graphs show the individual values of 39 patients treated with 0.075 to 0.9 mg Selectikine alone. Bars represent geometric mean values, with 95% CI. **B.** Frequency of GrB$^+$ Perforin$^+$ cells in NK gated cells on days 1 and 8 during the first and second treatment cycles. The box-and-whiskers graph shows geometric mean with 95% CI. * p < 0.05.

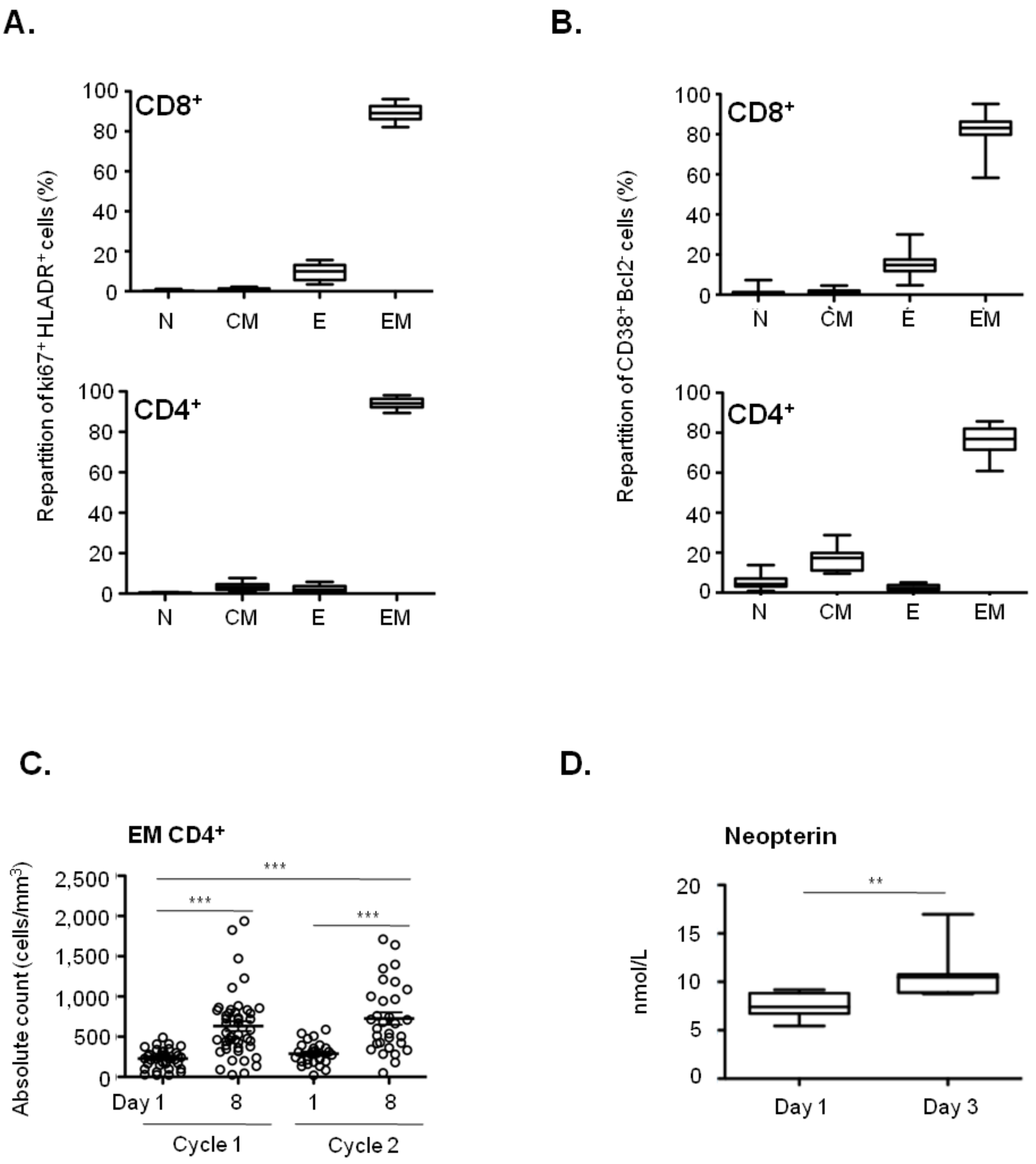

Supplementary Figure 2. T-cell proliferation and activation. **A.** Repartition (%) of proliferative cells (Ki67^{+} HLA-DR^{+}) and **B.** activated cells (Bcl2^{-} CD38^{+}) within subsets of CD4 and CD8 T-cells as defined by CCR7 and CD45RA expression, on day 8 during the first treatment cycle. N (Naïve), CCR7^{+} CD45RA^{+}; CM (Central Memory), CCR7^{+}

$CD45RA^-$; E (Effector), $CCR7^-$ $CD45RA^+$; EM (Effector Memory), $CCR7^-$ $CD45RA^-$. Box-and-whiskers graphs show geometric mean with 95% CI. **C.** Absolute counts (cell/mm^3) of Effector Memory (EM) CD4 T-cells on days 1 and 8 during the first and second cycles. The graph shows individual values of 39 treated patients. Bars represent the geometric mean with 95% CI. **D.** Plasma concentrations (ng/mL) of Neopterin measured on days 1 and 3 of each cycle of 39 treated patients. The graph shows the geometric mean per dose-group. ** $p \leq 0.01$, *** $p \leq 0.005$.

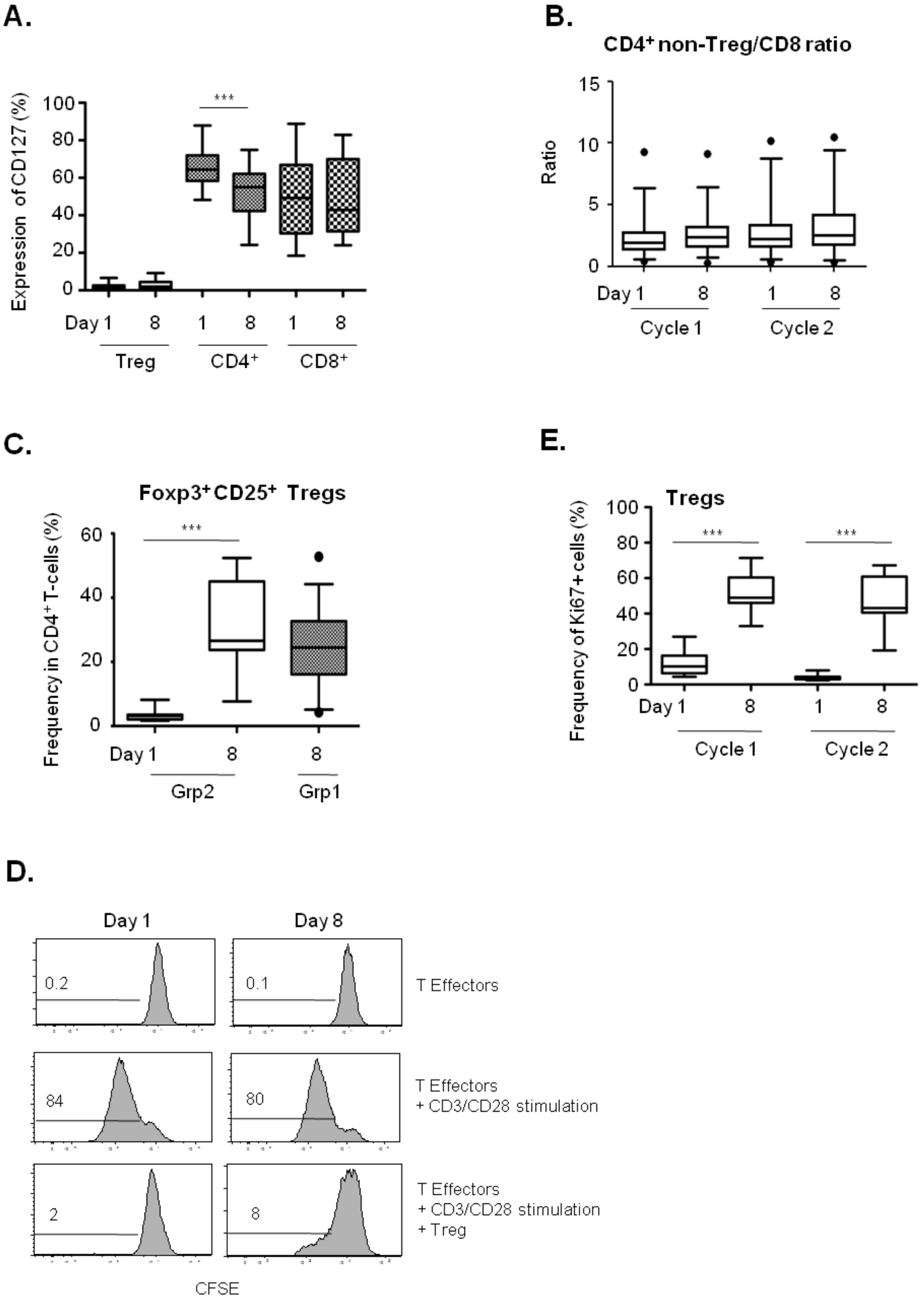
A.
Expression of CD127 (%)
100
80
60
40
20
0
***
Day 1
8
1
8
1
8
Treg
CD4+
CD8+
B.
CD4+ non-Treg/CD8 ratio
Ratio
15
10
5
0
Day 1
8
1
8
Cycle 1
Cycle 2
C.
Foxp3+CD25+ Tregs
Frequency in CD4+ T-cells (%)
60
40
20
0
***
Day 1
8
8
Grp2
Grp1
E.
Tregs
Frequency of Ki67+ cells (%)
100
80
60
40
20
0
***
***
Day 1
8
1
8
Cycle 1
Cycle 2
D.
Day 1
Day 8
0.2
0.1
T Effectors
84
80
T Effectors
+ CD3/CD28 stimulation
2
8
T Effectors
+ CD3/CD28 stimulation
+ Treg
CFSE

Supplementary Figure 3. Regulatory CD4 T-cells. **A.** Expression of CD127 (%) in Tregs ($CD4^+CD3^+Foxp3^+CD25^+$), CD4 T-cells ($CD4^+CD3^+$) and CD8 T-cells ($CD8^+CD3^+$) on days 1 and 8 during the first treatment cycle. The box-and-whiskers graph shows geometric mean with 95% CI. **B.** $CD4^+$ non-Treg cells/$CD8^+$ ratios on days 1 and 8 during the first and second treatment cycles. Box-and-whiskers graphs show geometric mean with 95% CI. **C.** Frequency (%) of Tregs ($Foxp3^+$ $CD25^+$) in $CD4^+$ T-cells on days 1 and 8 from patients pre-treated with low dose cyclophosphamide (CPA) before Selectikine administration (n= 9, group 2) compared to day 8 of the first cycle of patient group 1. Box-and-whiskers graphs show geometric mean with 95% CI. **D.** Typical CFSE staining representation of the inhibition by Treg cells of the proliferation of $CD4^+$ T-cells induced by *in vitro* stimulation with CD3/CD28 mAbs. Treg cells sorted on days 1 and 8 of the second cycle were simultaneously tested. **E.** Frequencies (%) of proliferative cells ($Ki67^+$) in $Foxp3^+$ Tregs on days 1 and 8 during the first and second treatment cycles. Box-and-whiskers graphs show geometric mean with 95% CI. *** $p \leq 0.005$.

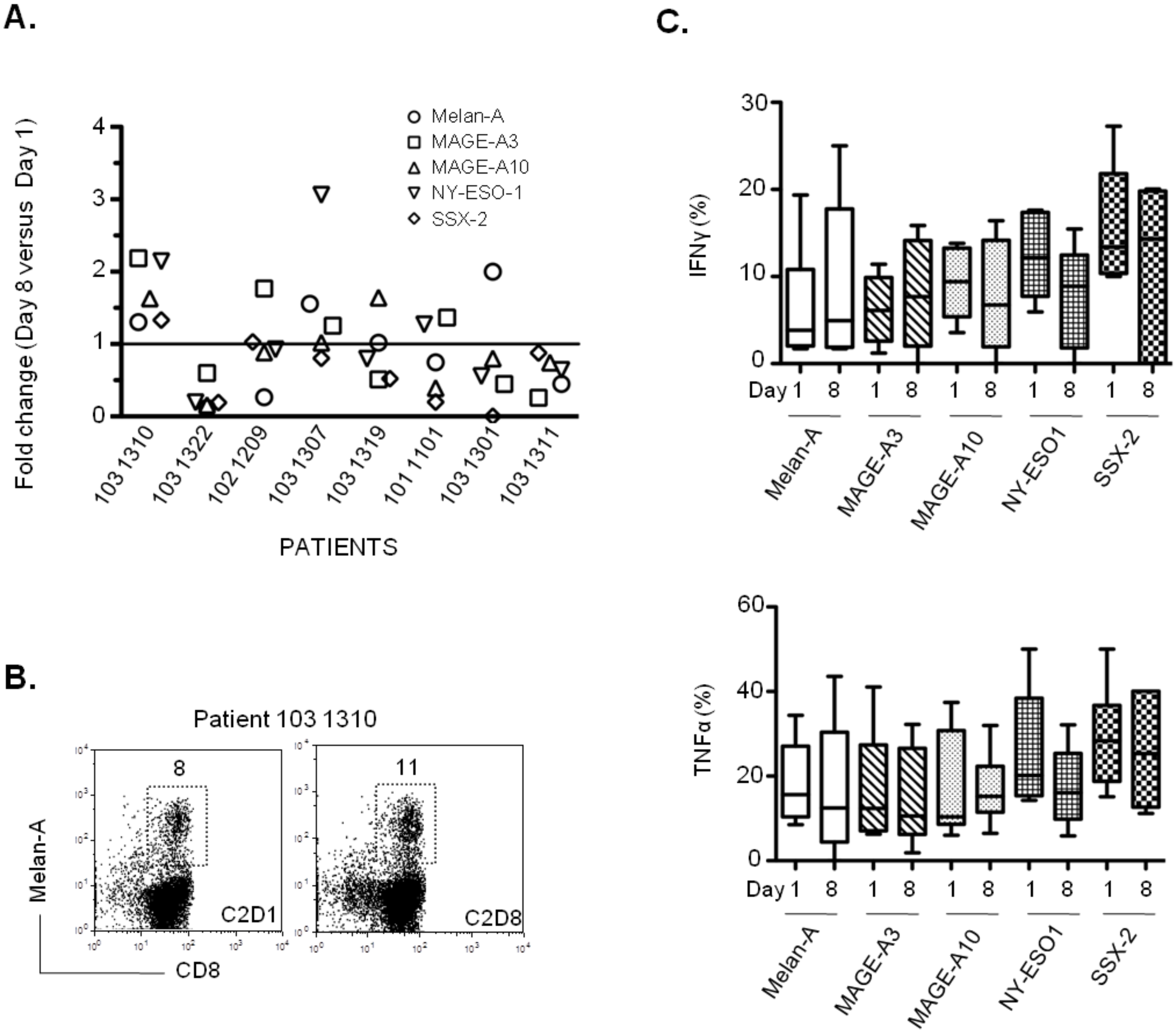

Supplementary Figure 4. Tumor antigen-specific T-cell responses. A. Fold change of percentages of cancer-testis specific $CD8^+$ T-cells on day 8 compared to day 1 of the second treatment cycle, from 8 HLA-A2 positive patients treated in group 1. Briefly, enriched $CD8^+$ T-cells from days 1 and 8 samples were cultured for one-week in presence of Melan-A, MAGE-A3, MAGE-A10, NY-ESO-1 and SSX-2 peptides, then stained with corresponding tetramers and CD8 mAb, and analyzed by FACS. **B.** Dot plots showing CD8 and Melan-A tetramer staining from patient 103 1310 after stimulation with Melan-A peptide (C2D1, cycle 2 day 1; C2D8, cycle 2 day 8). **C.**

Frequency (%) of IFNγ and TNFα positive cells within tumor antigen-specific $CD8^+$ T-cells after one-week peptide stimulation from blood samples collected on days 1 and 8 of the second cycle. Box-and-whiskers graphs show geometric mean with 95% CI from 8 HLA-A2 positive patients treated with 0.075 to 0.9 mg/kg Selectikine.

A.

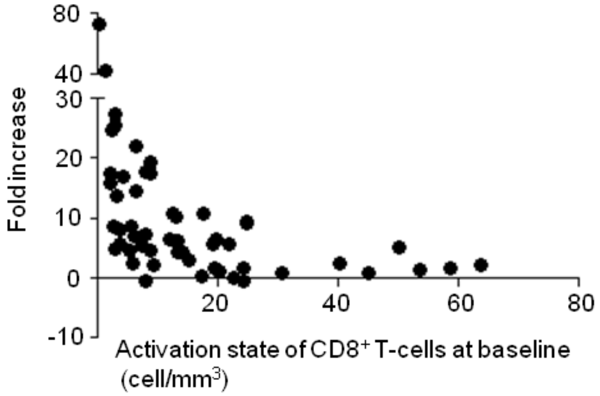

80
40
30
20
10
0
-10
20
40
60
80
Fold increase
Activation state of CD8+ T-cells at baseline (cell/mm3)

B.

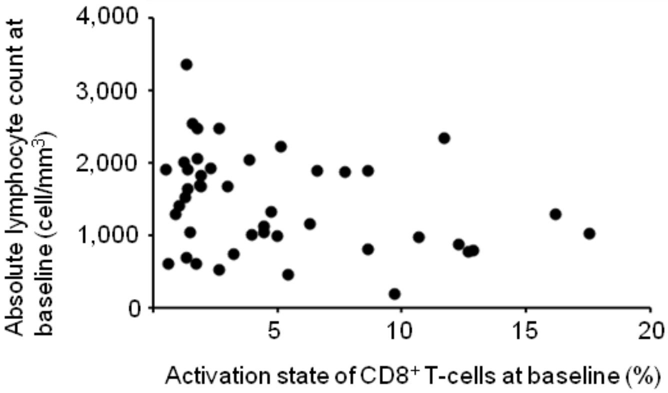

4,000
3,000
2,000
1,000
0
5
10
15
20
Absolute lymphocyte count at baseline (cell/mm3)
Activation state of CD8+ T-cells at baseline (%)

C.

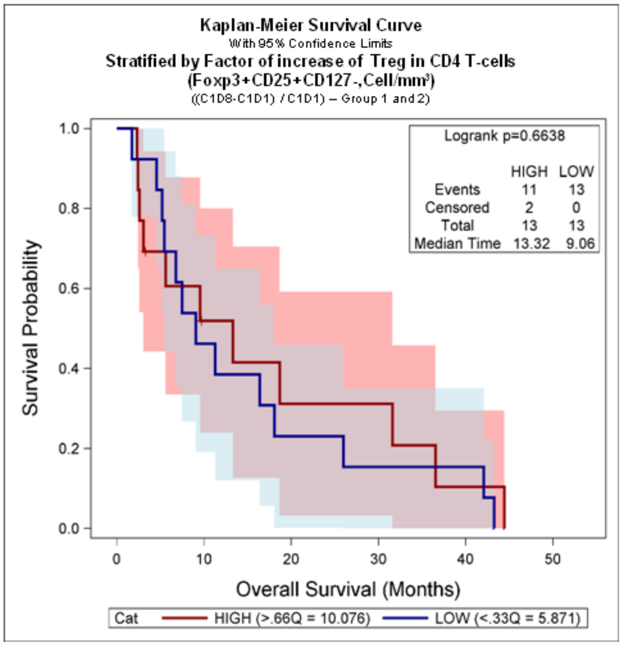

Kaplan-Meier Survival Curve
With 95% Confidence Limits
Stratified by Factor of increase of Treg in CD4 T-cells
(Foxp3+CD25+CD127-,Cell/mm3)
((C1D8-C1D1) / C1D1) – Group 1 and 2)
Logrank p=0.6638
HIGH LOW
Events 11 13
Censored 2 0
Total 13 13
Median Time 13.32 9.06
Survival Probability
1.0
0.8
0.6
0.4
0.2
0.0
0
10
20
30
40
50
Overall Survival (Months)
Cat
HIGH (>.66Q = 10.076)
LOW (<.33Q = 5.871)

Supplementary Figure 5. Activation state of CD8 T-cells. A. Fold increase of activated T-cells ($CD38^+$ $bcl2^-$ cells, cell/mm^3) after as compared to before treatment (treatment cycle 1), in relation to absolute count (cell/mm^3) of activated T-cells before treatment (at baseline). **B.** Percentages of activated T-cells (% $CD38^+$ $bcl2^-$ cells) at baseline compared to baseline lymphocyte counts (cell/mm^3). Data from group 1 and 2 are pooled. **C.** Kaplan-Meier Survival Curve with 95% confidence limits stratified by the fold of increase of Treg in $CD4^+$ T-cells ($Foxp3^+CD25^+CD127^-$, cell/mm^3) during first cycle of treatment, in patient groups 1 and 2.
